# 

**Published:** 1905-02-15

**Authors:** 


### ANNOUNCEMENTS.

THE Southern Branch of the National Dental Association holds its meeting February 21st to 24th, at Memphis, Tenn., in connection with the Tennessee State Dental Association. A strong and interesting program is offered and this should be a good meeting.

The Kentucky State Dental Association meets this year at Lexington, May 15th and 16th. A cordial invitation is extended to all practicing dentists to attend, and also to notify the Secretary of contributions in the way of papers or clinics. Dr. W. M. Randall, *Sec'y*,
Masonic Building, Louisville.

—✦—

Southwestern Michigan Dental Society.—The next meeting will be held in Kalamazoo, April 11th and 12th. This is one of the live, sociable societies that does good work and always has a good time. It is one of its unwritten laws that it will hold no meetings in a place whose police authorities will not give it perfect freedom to deport itself as the spirit moves, consequently its members always keep out of jail, and the people of the town know there has been something doing. A good program is already promised, and because of the well-known hospitality of the Kalamazoo fraternity, a splendid time is assured.

—✦—

National Dental Association—coming meeting—Section III. In preparation for the meeting of the National Dental Association at Buffalo, N. Y., July 25th to 28th, 1905, original papers and investigations are asked to be submitted upon the following topics: Oral surgery, anatomy, physiology, histology, pathology, etiology, hygiene, prophylaxis, materia medica, and allied subjects.

J. D. Patterson, *Chairman Sect. III*,

Kansas City, Mo.

